# The Phenomenology of Offline Perception: Multisensory Profiles of Voluntary Mental Imagery and Dream Imagery

**DOI:** 10.3390/vision9020037

**Published:** 2025-04-21

**Authors:** Maren Bilzer, Merlin Monzel

**Affiliations:** 1 Department of Psychology, Personality Psychology and Biological Psychology, University of Bonn, Kaiser-Karl-Ring 9, 53111 Bonn, Germany; maren.bilzer@uni-bonn.de; 2 Centre for Clinical Brain Sciences, University of Edinburgh, Edinburgh EH16 4SB, UK

**Keywords:** offline perception, phantom perception, voluntary mental imagery, dream imagery, multisensory imagery, dream recall, lucid dreaming

## Abstract

Both voluntary mental imagery and dream imagery involve multisensory representations without externally present stimuli that can be categorized as offline perceptions. Due to common mechanisms, correlations between multisensory dream imagery profiles and multisensory voluntary mental imagery profiles were hypothesized. In a sample of 226 participants, correlations within the respective state of consciousness were significantly bigger than across, favouring two distinct networks. However, the association between the vividness of voluntary mental imagery and vividness of dream imagery was moderated by the frequency of dream recall and lucid dreaming, suggesting that both networks become increasingly similar when higher metacognition is involved. Additionally, the vividness of emotional and visual imagery was significantly higher for dream imagery than for voluntary mental imagery, reflecting the immersive nature of dreams and the continuity of visual dominance while being awake and asleep. In contrast, the vividness of auditory, olfactory, gustatory, and tactile imagery was higher for voluntary mental imagery, probably due to higher cognitive control while being awake. Most results were replicated four weeks later, weakening the notion of state influences. Overall, our results indicate similarities between dream imagery and voluntary mental imagery that justify a common classification as offline perception, but also highlight important differences.

## 1. Introduction

“*Dreams, they feel real while we are in them, right? It’s only when we wake up that we realize that something was actually strange*”.

In Christopher Nolan’s science fiction film *Inception*, the protagonist Dominick Cobb raises a central philosophical question that is also discussed in research [1]: How can we ever be sure that we are awake and not dreaming? Dreaming is often perceived as an act of imagination [2,3], because, like imagining, it creates simulations of perception. Thus, the question can be extended: How can we ever be sure that we are actually perceiving external stimuli and not just imagining? Therefore, the present study aims to investigate the extent to which dream imagery and voluntary mental imagery are phenomenologically distinguishable and how they differ from sensory perceptions in terms of vividness by correlating the vividness ratings of the different sensory modalities for dream imagery and voluntary mental imagery, considering the roles of dream memory and lucid dreaming as moderators. Within this context, vividness refers to the measure of how close the mental image comes to an actual perception [4].

### 1.1. Dream Imagery and Voluntary Mental Imagery as Offline Perceptions

Dream imagery and voluntary mental imagery can both be understood as sensory representations in the absence of external stimuli [5,6,7]. Thus, they are often commonly classified as phantom [8] or offline perceptions [9]. An important difference is that voluntary mental imagery is generated voluntarily, whereas dreams mostly lack volitional control [5,7,8]. Neuronally, both types of imagery share the default mode network [5,10], and people who frequently recall their dreams have stronger activations in the default mode network while dreaming and being awake [11]. In addition, the loss of voluntary visual imagery may coincide with the loss of visual dream imagery as a result of damage to the hippocampus and disrupted visual pathway connections [12].

Voluntary mental imagery is thought to follow the same neural pathways as perception, albeit top-down. Accordingly, imagining functions like perception in reverse and starts a cascade beginning in frontal areas to the visual cortex [5]. Evidence for the assumption of voluntary mental imagery being a weaker form of perception is provided by visual imagery priming [13]. For example, ref. [14] demonstrated that imagining a stimulus influences which stimulus enters consciousness in a binocular rivalry task. Ref. [15] showed a priming effect of imagining colours on the perception of colours, and [16] as well as [17] found that mental imagery primes a visual search. Another study indicated that imagery resembles the characteristics of perceptions by showing that participants’ pupils dilated or constricted corresponding to the brightness of the imagined stimulus [18]. Moreover, visual mental imagery can be decoded using fMRI activity patterns in the visual cortices [19]. Ref. [20] showed that perceptual learning was possible through mental imagery alone. Furthermore, it was shown that people who were born blind were impaired in mental imagery [21]. Last, ref. [22] revealed that higher ratings of voluntary mental imagery vividness were associated with a smaller difference between perception and imagery in the activity patterns of visual areas.

The communalities between dreams and perception can also be elaborated. Ref. [23] applied the decoding of visual dream content using machine-learning models trained on stimulus-induced fMRI activity as a neural measure of the visual dream content. Images were assigned to specific fMRI activities based on activity during perception. The accuracy of decoding was confirmed by dream reports. Ref. [24] found that anterior cortical regions were activated during thought-like dream experiences and posterior cortical regions were activated during perceptual dream experiences with vivid sensory contents. Faces, for example, were accompanied by activation in the fusiform face area, and dreams with a specific spatial environment were associated with increased activity in the right posterior parietal cortex. Ref. [25] proposed that our eyes scan the dream scene during REM sleep, providing further evidence for the visual properties of dreams. Overall, these results indicate that dream imagery and voluntary mental imagery show many neural and behavioural commonalities with perception. Derived from this, we will examine the phenomenological overlap with regard to multisensory experiences.

### 1.2. Multisensory Profiles of Voluntary Mental Imagery and Dream Imagery

Voluntary mental imagery is most frequently investigated within the visual domain [26]. However, mental imagery is multisensory in nature and includes additional modalities, such as auditory, olfactory, gustatory, tactile, proprioceptive, and emotional imagery [27]. The vividness of mental imagery differs depending on the respective sensory system. Vividness is rated highest for visual impressions, followed by tactile and auditory impressions, whereas the ratings for emotional and proprioceptive impressions and especially for gustatory and olfactory impressions are lower [28]. The ratings vary to some extent; for example, ref. [29] found that mental imagery especially involves auditory imagery, but also that auditory and visual imagery occur more often in both imagination and dreams than gustatory, olfactory, and tactile imagery. Importantly, judgements of vividness correlate with the activation in associated modality-specific brain regions [30,31]. A left-lateralised modality-general network as well as modality-specific sensory areas could be identified [6,32].

Dream imagery is also multisensory in nature. Ref. [33] gives an overview of various studies of the predominant sensory modalities in dreams. Visual impressions were most common (100%), followed by auditory impressions (65%). In contrast, only 1% reported experiencing gustatory, olfactory, or tactile sensations [34]. A review [35] presents predominantly visual imagery, followed by frequent auditory impressions and less frequent gustatory, olfactory, vestibular, and tactile impressions. A study by [36] found less frequent reports of gustatory, olfactory, and tactile experiences than reports of visual, auditory, and kinaesthetic modalities. The subjects were thus divided into three groups: firstly, participants who reported experiencing mainly visual, auditory, and kinaesthetic imagery; secondly, a group who experienced multisensory imagery; and thirdly, a group who reported no sensory experience at all. The group with multisensory dream imagery also showed the highest ability to generate voluntary mental imagery while being awake, suggesting a common cognitive mechanism. Importantly, many of the aforementioned studies measured the frequency of various sensory experiences in dream imagery, whereas the vividness of dream imagery was less examined. We aim to explore the vividness of dream imagery to make it more comparable to the phenomenology of voluntary imagery.

### 1.3. The Continuity Hypothesis

The *continuity hypothesis* postulates a continuity between experiences while being awake and dreaming [37]. Studies indicate a continuity between the same conceptions and personal concerns, as well as between activities during dreaming and being awake [38]. Negative and positive daytime emotions influence dream emotions and vice versa [39]. Additionally, ref. [40] found similarities in cognitive and sensory qualities, suggesting the same cognitive–perceptual system being active during both experiences. Recent neurophysiological perspectives on the continuity hypothesis indicated similar underlying neuronal mechanisms and the same cognitive and emotional functioning across different states of consciousness [41].

A similar continuity hypothesis can be proposed for between perception and voluntary mental imagery. The *constructive episodic simulation hypothesis* (CESH) [42,43] postulates that remembering and imagining share the same characteristics and depend on constructive and simulative processes. When we remember a past experience, we access different independent elements from our memory to reconstruct a perceptual representation. When imaging something, a new representation is constructed from the same data base we use for remembering. Thus, our imagination is dependent on previous perceptions. Ref. [44] expands the CESH to the CESH+, including visual, auditory, gustatory, tactile, olfactory, and emotional sensory modalities. The same sensory brain areas are activated while generating episodic memory and voluntary mental imagery [6,45]. However, while remembering and imagining are mostly voluntary processes (excluding phenomena such as daydreaming, mind wandering, and intrusive memory), dream imagery is involuntary. Ref. [44] explains that dreaming also includes this constructive process but the elements to construct dream imagery can be retrieved without intention, referring to evidence provided by [46], who found that the dreams of patients with hippocampal damage include fewer episodic details, such as multisensory imagery.

Overall, it seems apparent that our sensory perceptions shape our voluntary sensory imagery as well as our sensory dream imagery in terms of memory. Therefore, it is conceivable that people who experience more sensations in one modality (e.g., a perfumer) develop both more voluntary mental imagery *and* dream imagery within this modality.

**Hypothesis 1:**  *There are significant positive correlations between the vividness ratings of different sensory modalities in voluntary mental imagery and the same sensory modalities in dream imagery*.

### 1.4. Dissociating Dream Imagery and Voluntary Mental Imagery

The conception of a common mechanism between voluntary mental imagery and dream imagery is challenged by the phenomenon of aphantasia, defined as a reduced or absent ability to produce voluntary mental imagery. Around 26.22% of people with aphantasia report a complete absence of multisensory imagery [47] while still being able to produce dream imagery [48]. However, they dream significantly less frequently and less vividly [47]. The fact that aphantasics can experience dreams may be reflected in a disturbance higher in the cascade of imagery generation rather than in sensory cortices [49]. Therefore, although sharing some neural substrates, dream imagery and voluntary mental imagery also rely on distinct mechanisms, leading to the following hypothesis:

**Hypothesis 2:**  *There are stronger positive correlations of different sensory modalities within voluntary mental imagery and within dream imagery (= monodomain correlations) than across voluntary mental imagery and dream imagery (= multidomain correlations)*.

Moreover, the studies presented above provided some evidence that certain sensory modalities, such as tactile, gustatory and olfactory imagery, occur very rarely or not at all in dreams. From the rare occurrence of these sensory modalities we would also assume lower vividness, since the vividness of mental imagery and frequent occurrence of mental imagery is often associated, at least while being awake, e.g., ref. [50]. For dreams, there is an association between dreams recall frequency and vividness of dream imagery [51]. Thus, we propose the following hypothesis:

**Hypothesis 3:**  *The vividness of tactile, gustatory, and olfactory imagery is lower for dream imagery than for voluntary mental imagery*.

A last difference between dream imagery and voluntary mental imagery is the “here and now” quality and immersive nature of dreams [1,52], leading to the appearance of reality until waking up [53]. According to [54], reality testing is missing while dreaming, since frontal regions are less activated, resulting in impaired executive functions, less top-down control, and deficient reality monitoring [7]. In contrast, imagination involves frontal and parietal regions to a higher extent [55,56], which enables cognitive control and reality monitoring [57]. This becomes especially apparent when looking at the affective component of dreams. Ref. [58] found that 74% of dreams contained emotions. While voluntary mental imagery also contains emotional representations [59], dreams are marked by immersion and hallucinatory qualities, amplifying the emotions and making them more intense [60]. Thus, one would not confuse a voluntarily imagined emotion with a real emotion, unlike in dreams.

**Hypothesis 4:**  *The vividness of emotional imagery is higher for dream imagery than for voluntary mental imagery*.

### 1.5. Further Influences

Although we argued for some differences between the vividness of voluntary mental imagery and the vividness of dream imagery, we also assumed some moderating factors that may attenuate the differences between both phenomena.

#### 1.5.1. Dream Recall

Despite experiencing sensory impressions, emotions, and thoughts during dreaming, we often do not remember them after waking up. Therefore, unsuccessful dream recall restricts the access to the phenomenology of dreams. The ability to recall a dream is therefore an important prerequisite for dream research and the only access to subjective dream experiences [61].

Dream recall frequency varies between and within individuals. On the one hand, trait factors were identified, such as interest in dreams, attitude towards dreams, personality factors, and socio-demographic variables. On the other hand, state factors were identified, such as poor sleep quality, frequent night waking, the presence of a mental illness, and stress [61,62]. Another reason for forgetting dreams can be a low dream quality [63]. According to [64], a higher capacity to produce visual imagery enriches the dream experience and promotes more frequent dream recall, which could also be demonstrated in a correlation between dream recall frequency and vividness of visual and tactile voluntary imagery [51]. However, it is not entirely clear whether people actually remember more vivid dreams or whether remembered dreams only appear more vivid due to more conscious processing.

Overall, since people lack access to the vividness of their dream imagery in the absence of dream recall, dream recall is most likely a moderator of the association between vividness of dream imagery and vividness of voluntary mental imagery. By considering dream recall as a moderator, the differences between the vividness of various sensory modalities in dream imagery and voluntary mental imagery should be reduced.

**Hypothesis 5:**  *The association between the vividness of voluntary mental imagery and vividness of dream imagery is moderated by the dream recall frequency*.

#### 1.5.2. Lucid Dreaming

In the same vein, lucid dreaming can be perceived as a moderating factor for differences between the vividness of voluntary mental imagery and the vividness of dream imagery. Typically, dreams are not accessible to metacognition. The exception are lucid dreams, in which dreamers gain the ability to reflect on their current state of dreaming [65]. At the same time, the dreamer remains in the physiological state of sleep. In an EEG study, ref. [66] showed increased activation during lucid dreaming in the right dorsolateral prefrontal cortex, the parietal lobe, bilateral frontopolar regions, and the precuneus, that is, in regions associated with metacognition; higher cognitive capacities; processing of one’s own thoughts, feelings, and actions; as well as experiences from the first-person perspective [67,68,69]. In contrast, these regions exhibit lower activity in non-lucid dreams [70]. Ref. [71] concludes that lucid dreaming is not a pure REM sleep phenomenon, but has characteristics of both REM sleep and the waking state and is thus to be understood as a hybrid state of consciousness. By understanding lucid dreams as a hybrid state, it can be argued for connections between the vividness of voluntary mental imagery and dream imagery. For example, ref. [72] demonstrated that the vividness of multisensory imagery during wakefulness predicts the frequency of lucid dreaming and [73] showed that lucid dreams are rated as more visual, auditory, and emotional than non-lucid dreams.

Overall, since dreamers gain executive control during lucid dreaming, lucid dreams are processed more deeply and experiences can be better remembered and reported, leading to a stronger association between the vividness of voluntary mental imagery and dream imagery.

**Hypothesis 6:**  *The association between the vividness of voluntary mental imagery and the vividness of dream imagery is moderated by the frequency of lucid dreams*.

#### 1.5.3. State Effects

Last, state effects on vividness ratings must be considered. Ref. [74] states that mental imagery vividness should not be understood as a stable maximum performance trait or an ability but as a dynamic performance trait. Accordingly, retest reliabilities are limited but still too high to define mental imagery vividness as an unstable state. Potential effects of variables like social desirability, self-confidence, training, and context on imagery ratings were considered. For instance, ref. [75] showed that contextual factors and movement during imagery had an enhancing effect on vividness ratings. By understanding imagery as a dynamic process, it becomes clear that studying the phenomenology of mental imagery requires multiple testing. Furthermore, it can be assumed that the vividness of dream imagery is also dynamic rather than stable and varies from dream to dream. For example, ref. [76] found several factors affecting the dream experience, such as the emotional intensity of current life events. Therefore, we tested our participants at two different measurement times to ensure that our effects were stable and not dependent on one specific point in time.

## 2. Materials and Methods

### 2.1. Participants

**Figure 1:**
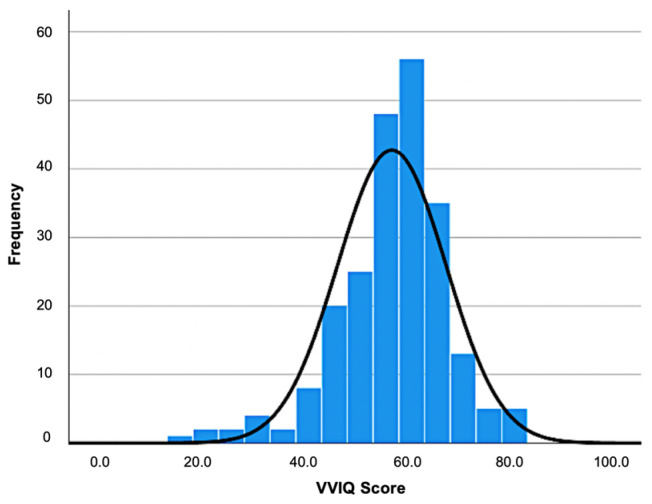
Distribution of VVIQ sum scores in the sample (*N* = 226). A normal distribution is superimposed over the empirical distribution.

The initial sample size consisted of *N* = 226 participants who completed measurement 1. Of those, *N* = 158 participants completed measurement 2. Participants were recruited by advertisement on university websites, social media, and word-of-mouth advertising. Inclusion criteria were legal age and reachability via e-mail. Among the sample, 3 participants were classified as aphantasics (VVIQ < 23; according to [48]) and 9 were classified as hyperphantasics (VVIQ > 75; [48]). The remaining participants reached a mean score on the VVIQ of *M* = 57.12 (*SD* = 10.55). The VVIQ sum scores followed a normal distribution (see Figure 1). The age ranged from 18 to 59 years (*M* = 25.51; *SD* = 8.46). The sample was composed of 186 females (82.3%), 39 males (17.3%) and 1 participant of another gender (0.4%). The level of education was generally high (7% secondary school, 70.4% A-levels, 21.2% university degree, 0.4% doctoral degree, and 0.9% other).

### 2.2. Materials

The *Vividness of Visual Imagery Questionnaire* (VVIQ) [4] was used to measure visual voluntary mental imagery. Four different visual scenarios are described and the participants are instructed to produce the corresponding mental images. They are then asked to rate how vivid these are, that is, how closely they resemble their actual perception. There are 16 items, which are to be rated on a five-point Likert scale from 0 (“*No image at all, you only know that you are thinking of the object*”) to 5 (“*Perfectly clear and as vivid as normal vision*”). An example for an item is “Visualize a rising sun. Consider carefully the picture that comes before your mind’s eye. A rainbow appears”.

The vividness of multisensory voluntary mental imagery was measured by means of the *Plymouth Sensory Imagery Questionnaire* (PSI-Q) [28]. The PSI-Q includes items for the following sensory modalities: vision, sound, smell, taste, touch, bodily sensation, and emotional feeling. The questionnaire consists of seven sets of five items, each regarding one modality. The participants were instructed to rate how similar their mental imagery was to actual perception. An example for an item is “Imagine the sound of an ambulance siren”, which had to be rated on a 11-point scale anchored by 0 (“*No image at all*”) and 10 (“*As vivid as in real life*”).

To measure the vividness of multisensory imagery in the state of dreaming, the PSI-Q was adapted. The new form of the PSI-Q included one item for each modality concerning dreams. The instruction was to rate how similar the sensory modalities occurring in the participant’s latest dreams were to actual perception on a 11-point scale from 0 (“*No image at all*”) to 10 (“*As vivid as in real life*”).

In order to examine additional features of dreaming, the *Mannheim Dream Questionnaire* (MADRE) [77] was applied. A seven-point scale was used to elicit the dream recall frequency (0 = “*never*”, 1 = “*less than once a month*”, 2 = “*about once a month*”, 3 = “*about 2 to 3 times a month*”, 4 = “*about once a week*”, 5 = “*several times a week*”, 6 = “*almost every morning*”). The lucid dreaming frequency was measured on an eight-point scale (0 = “*never*”, 1 = “*less than once a year*”, 2 = “*about once a year*”, 3 = “*about two to four times a year*”, 4 = “*about once a month*”, 5 = “*about two to three times a month*”, 6 = “*about once a week*”, 7 = “*several times a week*”), accompanied by a definition of lucid dreaming. The poles of the MADRE were reversed before the analyses to obtain an understandable interpretation. Otherwise, for example, a low score would have indicated frequent dream recall. As the MADRE does not have metric scale levels, the following three groups were formed for moderation analyses, *less than once a month*, *at least once a month* and at *least once a week*, to ensure sufficiently large subgroups.

### 2.3. Procedure

The study was conducted online via the software *SoSci Survey* (Version 3.2.50) [78]. The questionnaire was suitable for use with laptops, tablets, as well as smartphones. An incentive to participate was given by offering feedback regarding the ability to create multisensory imagery based on the PSI-Q and by offering student credit (0.75 subject hours) for psychology students. The study contained two measurements taken four weeks apart. The participants were automatically reminded by e-mail to take part in the second measurement. The participation in each measurement took around 10 to 15 min. Measurement 1 included the VVIQ, the original PSI-Q, the dream PSI-Q, and the three items of the MADRE described above. Measurement 2 included the VVIQ, the original PSI-Q, and the dream PSI-Q. Before the survey started, participants were informed about anonymity, voluntariness, and had to provide informed consent, followed by questions regarding sociodemographic data as gender, age, and education.

### 2.4. Statistical Analyses

**Figure 2:**
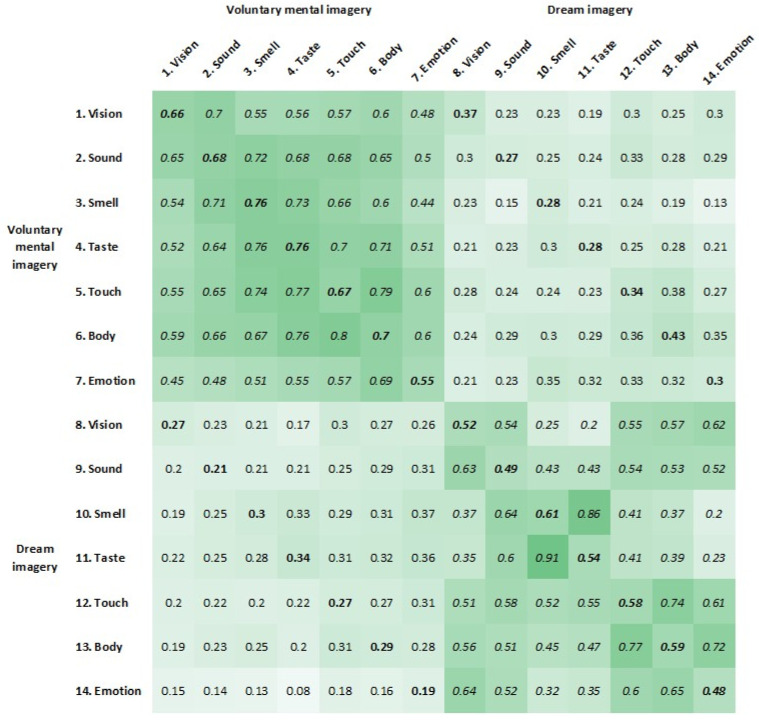
Intercorrelations of study variables in the form of a heat map. The results for measurement time 1 (*N*_1_ = 221) are shown below the diagonal, and the results for measurement time 2 (*N*_2_ = 156) are shown above the diagonal. Monodomain correlations are depicted in italics. Monosense–multidomain correlations are depicted in bold. Retest reliabilities are depicted in bold and italics. The darker the shade of green, the higher the correlation. The underlying multisense–multidomain matrix can be found in the Supplementary Materials. All correlations > 0.15 were significant.

Unless otherwise stated, analyses were conducted using SPSS (Version 27.0) [79]. Retest reliabilities and intercorrelations between the vividness of different sensory modalities within and between voluntary mental imagery and dream imagery were assessed via Pearson’s correlations and presented in a heat map reflecting a multisense–multidomain matrix following conventional multitrait–multimethod matrices. The term “sense” is used to denote the sensory modality and “domain” refers to voluntary mental imagery or dream imagery. For correlation comparisons, correlations were mean-aggregated into monosense–monodomain correlations (i.e., averaging retest correlations within voluntary mental imagery and within dream imagery), monosense–multidomain correlations (i.e., averaging correlations of single sensory modalities across voluntary mental imagery and dream imagery), multisense–monodomain correlations (i.e., averaging correlations across sensory modalities within voluntary mental imagery and within dream imagery), and multisense–multidomain correlations (i.e., averaging correlations across sensory modalities across voluntary mental imagery and dream imagery) using Fisher’s z-transformations, before being compared with each other using the online tool *Psychometrica* [80]. An additional network analysis was performed using JASP 0.16.2 [81] to assess the sparsity of the total model, as well as for voluntary mental imagery and dream imagery separately. A sparse network has fewer links than a denser network. Thus, a higher sparsity for the total model than for both individual models would indicate two separate networks. Additional correlation comparisons between dream recall and lucid dreaming subgroups were calculated to assess moderation effects. Paired *t*-tests were calculated to assess differences in the vividness of multisensory imagery between voluntary mental imagery and dream imagery. To cross-validate our results, all analyses were performed separately for both measurement times. Due to the larger sample size, only the analyses for measurement time 1 are reported in the main text (with the exception of the intercorrelations in Figure 2 to assess retest reliabilities), whereas the analyses for measurement time 2 can be found in the Supplementary Materials. Deviations between the two measurement times are highlighted.

## 3. Results

### 3.1. Associations Between the Vividness of Voluntary Mental Imagery and Dream Imagery

**Figure 3:**
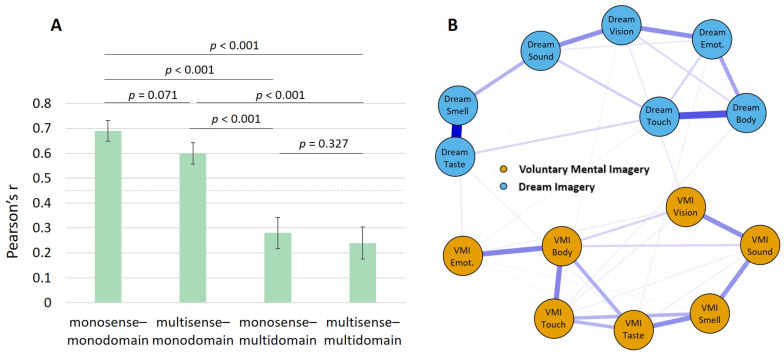
(**A**) Correlation comparisons for the different configurations of the multisense–multidomain matrix. The dotted line indicates the average correlation across all senses and domains. (**B**) Network graph including the subnetworks for the vividness of multisensory voluntary mental imagery (orange) and the vividness of multisensory dream imagery (blue).

The associations of vividness between the different sensory modalities within and between voluntary mental imagery and dream imagery are depicted in Figure 2. The main diagonal provides evidence for satisfactory retest reliabilities in all sensory modalities for both voluntary mental imagery (ranging from 0.55 to 0.76) and dream imagery (ranging from 0.48 to 0.61). Monosense–multidomain correlations are depicted in bold, indicating significant positive correlations between the vividness ratings of different sensory modalities in voluntary mental imagery and the same sensory modalities in dream imagery (Hypothesis 1). As indicated by the darker shades of green in the upper left and lower right quadrants in comparison to the upper right and lower left quadrants, correlations within voluntary mental imagery and within dream imagery, *r* = 0.61, were bigger than across the domains, *r* = 0.25, *z* = 4.30, *p* < 0.001 (Hypothesis 2). Correlation comparisons between all different configurations of the multisense–multidomain matrix can be found in Figure 3A.

Figure 3B depicts the associations between the several modalities of voluntary mental imagery and dream imagery in a network graph using an EBICglasso estimator. In comparison to the sparsity of the total model (= 0.54), the individual models for voluntary mental imagery (*sparsity* = 0.14) and dream imagery (*sparsity* = 0.24) were a lot denser.

**Figure 4:**
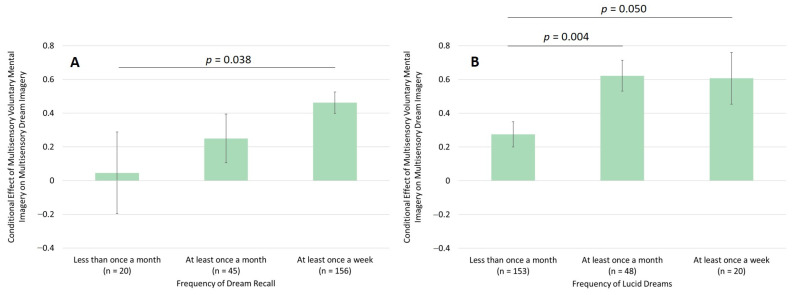
Conditional effects of multisensory voluntary mental imagery vividness on multisensory dream imagery vividness by (**A**) the frequency of dream recall and (**B**) frequency of lucid dreams.

Importantly, the association between the vividness of voluntary mental imagery and vividness of dream imagery was moderated by dream recall, as shown by a significantly higher correlation between the vividness of voluntary mental imagery and the vividness of dream imagery when participants recalled their dreams at least once a week, *r* = 0.46, in contrast to less than once in a month, *r* = 0.05, *z* = 1.78, *p* = 0.038 (see Figure 4A) (Hypothesis 5). The same was true for the frequency of lucid dreams: The correlation increased when participants had lucid dreams at least once in a month, *r* = 0.62, in contrast to less than once in a month, *r* = 0.28, *z* = 2.62, *p* = 0.004 (see Figure 4B) (Hypothesis 6). The moderation effect of lucid dreaming could be replicated at measurement time 2, whereas the moderation effect of dream recall was only found descriptively at measurement time 2.

### 3.2. Mean Differences Between Voluntary Mental Imagery and Dream Imagery

**Figure 5:**
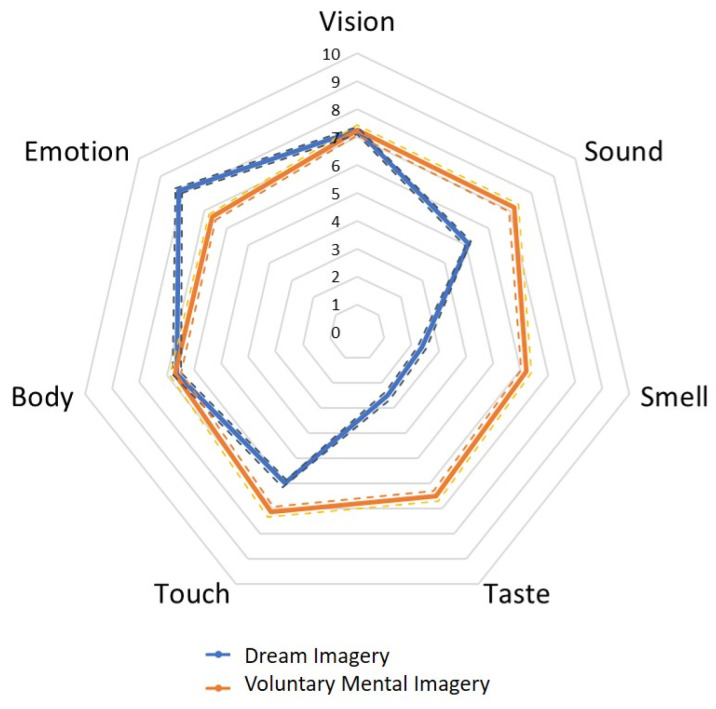
Means (solid lines) and standard errors (dotted lines) for multisensory voluntary mental imagery profiles (orange) and dream imagery profiles (blue).

Although multisense–monodomain correlations within voluntary mental imagery, *r* = 0.63, and within dream imagery, *r* = 0.55, did not differ significantly, *z* = 1.29, *p* = 0.099, the mean differences between the domains remain to be investigated. While the vividness of emotional imagery was bigger for dream imagery than for voluntary mental imagery, *t*(220) = 8.36, *p* < 0.001, *d* = 0.56 (Hypothesis 4), the vividness of auditory, *t*(220) = 9.39, *p* < 0.001, *d* = 0.63; olfactory, *t*(220) = 17.93, *p* < 0.001, *d* = 1.20; gustatory, *t*(220) = 18.99, *p* < 0.001, *d* = 1.28; and tactile imagery, *t*(220) = 4.91, *p* < 0.001, *d* = 0.33, were smaller for dream imagery (Hypothesis 3). For visual imagery, *t*(220) = 0.20, *p* = 0.984, *d* < 0.01, and proprioceptive imagery, *t*(220) = 0.22, *p* = 0.823, *d* = 0.02, no significant differences were found. Means and standard errors are depicted in Figure 5. At measurement time 2, aggregated multisense–monodomain correlations within voluntary mental imagery, *r* = 0.62, were significantly bigger than the aggregated multisense–monodomain correlations within dream imagery, *r* = 0.46, *z* = 1.99, *p* = 0.023. This could indicate more pronounced domain general processes in voluntary mental imagery than in dream imagery. Last, the vividness of visual imagery was higher in dream imagery than in voluntary mental imagery, *t*(155) = 2.08, *p* = 0.039, *d* = 0.17.

## 4. Discussion

Since the vividness of mental imagery is considered a dynamic trait [74], possible state influences on the vividness of voluntary mental imagery and vividness of dream imagery were considered by investigating them at two measurements four weeks apart. Evidence for satisfactory retest reliabilities was found in all sensory modalities for both the vividness of voluntary mental imagery and dream imagery. As most results were replicated at measurement time 2, both measurements are considered together in the following section.

All monosense–multidomain correlations were significant at measurement times 1 and 2 (acceptance of Hypothesis 1). This reflects the neurophysiological overlap between both processes and is consistent with findings that have shown that a loss of visual voluntary mental imagery due to brain damage can also lead to a loss of visual dream experience [12]. It is also consistent with the CESH+, which states that multisensory elements of episodic information must be recombined to produce voluntary and non-voluntary mental imagery [44]. Thus, in addition to the already known neural similarities, our results suggest a phenomenological overlap, supporting a common categorisation of voluntary mental imagery and dream imagery as offline perceptions [9]. The similarities in sensory qualities can also be interpreted as evidence for similar cognitive–perceptual functions across different states of consciousness, which supports the continuity hypothesis of being awake and dreaming [40,81,82,83,84,85].

Next, the monodomain correlations were significantly bigger than the multidomain correlations at measurement times 1 and 2 (acceptance of Hypothesis 2), providing evidence for additional domain-specific processes involved in voluntary mental imagery and dream imagery. In line with this, multisense–multidomain correlations as well as monosense–multidomain correlations were significantly smaller than multisense–monodomain correlations and monosense–monodomain correlations, which can be attributed to the fact that the first correlations were calculated across domains. In contrast, multisense–multidomain correlations were not significantly different from monosense–multidomain correlations and monosense–monodomain correlations were not significantly different from multisense–monodomain correlations, indicating that the correlations were less affected by differences in the sensory modalities. Importantly, the correlations across domains were moderated by dream recall frequency at measurement time 1 (partial acceptance of Hypothesis 5) and by the frequency of lucid dreaming at measurement times 1 and 2 (acceptance of Hypothesis 6). Correlations between the vividness of voluntary mental imagery and vividness of dream imagery were significantly higher when dreams were recalled at least once a week in contrast to less than once a month. This confirms the crucial role of dream recall, since dream recall is a prerequisite to gain access to subjective dream experiences and to investigate the sensory modalities involved in dream imagery. While mental imagery vividness was assessed immediately after participants created the mental image, dream vividness was assessed with a delay, considering the participants’ latest dreams.

With regard to lucid dreaming, correlations between the vividness of voluntary imagery and vividness of dream imagery were significantly higher when lucid dreams occurred at least once a month. A higher degree of executive control would be a possible explanation, indicating that a certain degree of executive control and metacognition is present in lucid dreams, but not in non-lucid dreams [86]. The generation of dream imagery in lucid dreaming is therefore more similar to voluntary mental imagery. Reference can be made to [2] which states that dreams are fundamentally subject to the will and, despite the lack of volitional control in dreams, the dreamer can in principle become conscious of his dreaming state. As postulated by [71], lucid dreams can be understood as a hybrid state that once again emphasizes the continuity of consciousness between being awake and dreaming.

Interestingly, the moderation effect of the frequency of lucid dreaming was found at both measurement times, while no significant moderation effect of the frequency of dream recall was found at measurement time 2, although it remained descriptively visible. One explanation could be that the effect of lucid dreaming is more stable, as lucid dreaming involves stronger executive control, which leads to more valid dream reports. Another explanation could be that the frequency of dream recall is more variable than the frequency of lucid dreams due to state influences [61,87]. Unfortunately, we only recorded the frequency of dream recall at measurement time 1, which is why the same value could be invalid at measurement time 2.

Despite the stark associations between the vividness of dream imagery and the vividness of voluntary mental imagery, differences were found in the level of vividness of individual sensory modalities. The vividness of emotional imagery was significantly higher for dream imagery than for voluntary mental imagery at measurement time 1 and 2 (acceptance of Hypothesis 4). This can be explained by the immersive and hallucinatory nature of dreams [60]. While dreaming, emotions are experienced as real, unlike when one tries to imagine a certain emotion and is aware of doing so. The results correspond with previous studies on the affective colouring of dreams, which found that dreams often contain intense emotions, e.g., ref. [58], probably due to consolidation of emotional memory during REM sleep [88].

In contrast to emotional imagery, the vividness of auditory, olfactory, gustatory, and tactile imagery was significantly smaller for dream imagery compared to voluntary mental imagery at both measurement times (acceptance of Hypothesis 3), probably due to higher cognitive control during wakefulness [7]. This is consistent with previous findings showing less frequent reports of gustatory, olfactory, and tactile imagery in dreams, e.g., ref. [36]. On the other hand, at least at measurement time 2, the vividness of visual imagery was higher in dream imagery than in voluntary mental imagery. This is in line with previous studies showing that dreams primarily contain visual imagery [35]. One explanation for the dominance of visual imagery during dreaming could be the continuity hypothesis. Since the visual system is the most important sensory system while being awake, it follows that it is also the most important sensory modality during dreaming (in combination with the immersive quality of dreams [1]). In the same vein, auditory, olfactory, gustatory, and tactile sensations are less pronounced when awake *and* dreaming, e.g., refs. [28,36]. This view is also supported by neuropsychological accounts of the continuity hypothesis that indicate similarities in cognitive and sensory qualities across different states of consciousness [41]. Thus, these results can be regarded as an argument in favour of a continuity between voluntary mental imagery and dream imagery as well.

Finally, the network graphs were denser when voluntary mental imagery and dream imagery were considered separately, indicating that multiple sensory impressions within one state of consciousness are phenomenologically more similar than across both states of consciousness, confirming the other results.

### 4.1. Implications

Our results suggest that the concept of offline perceptions can be justified on a phenomenological level due to similar multisensory experiences during voluntary mental imagery and dream imagery. We found correlations in terms of vividness in addition to previous results regarding the frequency of specific sensory modalities or shared neural activity. Consequently, certain insights gained from dream imagery or voluntary mental imagery should be transferable from one domain to the other, at least theoretically. Both forms of imagery can be considered as perception-like experiences in the absence of external stimuli. According to this definition, additional mental phenomena can be grouped in this category, such as daydreams and hallucinations [89]. These phenomena should be subject to further investigation, examining neural and phenomenological similarities. Ref. [7] associated hallucinations with dreams and imagination, while [54] suggested that daydreams are particularly similar to voluntary mental imagery. In future studies, a multisensory approach should continue to be used to investigate the relationships of individual sensory modalities in other forms of offline perceptions.

Importantly, the overlap of imagination, dreaming, and perception is of practical relevance to the clinical field. Mental imagery is important in several mental disorders such as PTSD, depression, and nightmare disorders [83,90]. Imagination techniques can reduce dysfunctional mental imagery [91], and nightmares can be treated through approaches such as *Imagery Rehearsal Therapy* [82,92]. These methods are supported by our finding of a strong phenomenological overlap between voluntary mental imagery and dream imagery. Moreover, in line with our results, lucid dreams are proposed as the next step in positively influencing nightmares [93], as they allow for greater cognitive control and are even more similar to dream imagery than voluntary mental imagery while being awake.

### 4.2. Strengths and Weaknesses

One methodological strength of the present study is the use of the PSI-Q [28], which made it possible to go beyond the assessment of visual imagery, on which research was strongly focused in the past, neglecting other sensory modalities. Analogous to the PSI-Q, a dream questionnaire was developed for the present study. Since the content of dreams cannot be predicted, the dream version of the PSI-Q contained only one general item per modality instead of several items referring to specific dream content. While assessments via single items can be criticized, the questionnaire proved to be retest reliable across both measurement times, further validating our results. However, it should be criticized that the MADRE items concerning dream recall and lucid dreaming were only assessed at the first measurement time. Hence, there was no information about the frequency of dream recall and lucid dreams at the second measurement time. It should also be mentioned that the vividness of voluntary mental imagery and dream imagery was assessed via self-reports, which are subject to response biases. Participants could have inferred their vividness of dream imagery from their vividness of voluntary mental imagery and vice versa or could have generalized from one sensory modality to another. However, despite the possibility of such generalization effects, differences in the vividness levels and correlations were found, rendering our design sufficient for uncovering the expected results. Last, we inferred a link between lucid dreaming and executive control to explain the moderation effect of lucid dreaming. Future research should aim to measure executive functions directly to strengthen our interpretation that executive control might explain the phenomenological overlap between voluntary mental imagery and imagery in lucid dreams.

## 5. Conclusions

Overall, two separate imagery networks were found, which became increasingly similar after taking dream recall and lucid dreaming into account. On a phenomenological level, this favours a common classification of voluntary mental imagery and dreams as stimulus-independent offline perceptions, using sensory similarity to perception as a criterion. However, even though both phenomena can be categorised as offline perceptions, they are still distinguishable due to other features, such as immersion and executive control.

## Data Availability

The data that support the findings of this study are openly available at https://osf.io/ubkv6/?view_only=33331f272a7f4304a7e82cd62393c4fe (accessed on 13 April 2025).

## Supplementary Materials

The following supporting information can be downloaded at https://www.mdpi.com/article/10.3390/vision9020037/s1, Table S1. Intercorrelations of study variables in form of a multisense–multidomain matrix disaggregated by measurement time.

## Abbreviations

The following abbreviations are used in this manuscript:

CESH	Constructed Episodic Simulation Hypothesis
VVIQ	Vividness of Visual Imagery Questionnaire
PSI-Q	Plymouth Sensory Imagery Questionnaire
MADRE	Mannheim Dream Questionnaire
