# Supplementary material for: The Phenomenology of Offline Perception: Multisensory Profiles of Voluntary Mental Imagery and Dream Imagery
Source: Vision (Basel). 2025 Apr 21;9(2):37. doi: 10.3390/vision9020037 (PMC12015918; doi:10.3390/vision9020037)
Supplement: Supplementary file 1 [file vision-09-00037-s001.zip › vision-3568760-supplementary.pdf]

Supplemental Material

Table S1

Intercorrelations of study variables in form of a multisense-multidomain-matrix disaggregated by measurement time.

		Voluntary mental imagery							Dream imagery						
		1	2	3	4	5	6	7	8	9	10	11	12	13	14
Voluntary mental imagery	1. Vision	___.66***___	_.70***_	_.55***_	_.56***_	_.57***_	_.60***_	_.48***_	__.37***__	.23**	.23**	.19*	.30***	.25**	.30***
	2. Sound	_.65***_	___.68***___	_.72***_	_.68***_	_.68***_	_.65***_	_.50***_	.30***	__.27***__	.25**	.24**	.33***	.28***	.29***
	3. Smell	_.54***_	_.71***_	___.76***___	_.73***_	_.66***_	_.60***_	_.44***_	.23**	.15	__.28***__	.21**	.24**	.19*	.13
	4. Taste	_.52***_	_.64***_	_.76***_	___.76***___	_.70***_	_.71***_	_.51***_	.21**	.23**	.30***	__.28***__	.25**	.28***	.21**
	5. Touch	_.55***_	_.65***_	_.74***_	_.77***_	___.67***___	_.79***_	_.60***_	.28***	.24**	.24**	.23**	__.34***__	.38***	.27***
	6. Body	_.59***_	_.66***_	_.67***_	_.76***_	_.80***_	___.70***___	_.60***_	.24**	.29***	.30***	.29***	.36***	__.43***__	.35***
	7. Emotion	_.45***_	_.48***_	_.51***_	_.55***_	_.57***_	_.69***_	___.55***___	.21**	.23**	.35***	.32***	.33***	.32***	__.30***__
Dream imagery	8. Vision	__.27***__	.23***	.21***	.17*	.30***	.27***	.26***	___.52***___	_.54***_	_.25**_	_.20*_	_.55***_	_.57***_	_.62***_
	9. Sound	.20**	__.21**__	.21**	.21**	.25***	.29**	.31**	_.63***_	___.49***___	_.43***_	_.43***_	_.54***_	_.53***_	_.52***_
	10. Smell	.19**	.25***	__.30***__	.33***	.29***	.31***	.37***	_.37***_	_.64***_	___.61***___	_.86***_	_.41***_	_.37***_	_.20*_
	11. Taste	.22**	.25***	.28***	__.34***__	.31***	.32***	.36***	_.35***_	_.60***_	_.91***_	___.54***___	_.41***_	_.39***_	_.23**_
	12. Touch	.20**	.22**	.20**	.22**	__.27***__	.27***	.31***	_.51***_	_.58***_	_.52***_	_.55***_	___.58***___	_.74***_	_.61**_
	13. Body	.19**	.23***	.25***	.20**	.31***	__.29***__	.28***	_.56***_	_.51***_	_.45***_	_.47***_	_.77***_	___.59***___	_.72**_
	14. Emotion	.15*	.14*	.13*	.08	.18**	.16*	__.19**__	_.64***_	_.52***_	_.32***_	_.35***_	_.60***_	_.65***_	___.48***___

Note. The results for measurement time 1 (N = 221) are shown below the diagonal. The results for measurement time 2 (N = 156) are shown above the diagonal. Monosense-multidomain-correlations are depicted in bold. Mulitsense-monodomain-correlations are depicted in italic. Retest reliabilities are depicted in bold and italic. * $p < .05$, ** $p < .01$, *** $p < .001$.

Measurement time 2

Associations between the vividness of voluntary mental imagery and dream imagery

The associations of vividness between the different sensory modalities within and between voluntary mental imagery and dream imagery at measurement time 2 are also depicted in Figure 2. As at measurement time 1, monodomain-correlations, $r = .58$, were significantly bigger than multidomain-correlations, $r = .27$, $z = 3.37$, $p < .001$. In comparison to the total model (= 0.48), the individual models for voluntary mental imagery (*sparsity* = 0.19) and dream imagery (*sparsity* = 0.24) were a lot denser. With regard to the moderation effects, the association between vividness of voluntary mental imagery and dream imagery only increased descriptively when frequency of dream recall was used as a moderator. For frequency of lucid dreams, the moderation effect was replicated, showing a higher correlation when people had lucid dreams at least once a month, $r = .66$, in contrast to less than once a month, $r = .37$, $z = 2.03$, $p = .021$ (see Figure 4b).

Mean differences between voluntary mental imagery and dream imagery

In contrast to measurement 1, the aggregated multisense-monodomain-correlations within voluntary mental imagery, $r = .62$, were significantly bigger than the aggregated multisense-monodomain-correlations within dream imagery, $r = .46$, $z = 1.99$, $p = .023$. Means and standard errors are depicted in Figure 5b. As at measurement time 1, vividness of emotional imagery was higher for dream imagery than for voluntary mental imagery, $t(155) = 7.77$, $p < .001$, $d = 0.62$. Results for vividness of auditory, $t(155) = 6.65$, $p < .001$, $d = 0.53$, olfactory, $t(155) = 16.26$, $p < .001$, $d = 1.30$, gustatory, $t(155) = 16.75$, $p < .001$, $d = 1.34$, tactile, $t(155) = 5.01$, $p < .001$, $d = 0.40$, and proprioceptive imagery, $t(155) = 0.10$, $p = .919$, $d = 0.01$, were replicated as well. In contrast to measurement time 1, vividness of visual imagery was higher in dream imagery than in voluntary mental imagery, $t(155) = 2.08$, $p = .039$, $d = 0.17$.
